# Crosstalk Between CD11b and Piezo1 Mediates Macrophage Responses to Mechanical Cues

**DOI:** 10.3389/fimmu.2021.689397

**Published:** 2021-09-22

**Authors:** Hamza Atcha, Vijaykumar S. Meli, Chase T. Davis, Kyle T. Brumm, Sara Anis, Jessica Chin, Kevin Jiang, Medha M. Pathak, Wendy F. Liu

**Affiliations:** ^1^Department of Biomedical Engineering, University of California, Irvine, Irvine, CA, United States; ^2^The Edwards Lifesciences Center for Advanced Cardiovascular Technology, University of California, Irvine, Irvine, CA, United States; ^3^Sue and Bill Gross Stem Cell Research Center, University of California, Irvine, Irvine, CA, United States; ^4^Department of Physiology and Biophysics, University of California, Irvine, Irvine, CA, United States; ^5^Department of Chemical Engineering and Materials Science, University of California, Irvine, Irvine, CA, United States; ^6^Department of Molecular Biology and Biochemistry, University of California, Irvine, Irvine, CA, United States

**Keywords:** macrophage, stretch, integrin, piezo1, inflammation

## Abstract

Macrophages are versatile cells of the innate immune system that perform diverse functions by responding to dynamic changes in their microenvironment. While the effects of soluble cues, including cytokines and chemokines, have been widely studied, the effects of physical cues, including mechanical stimuli, in regulating macrophage form and function are less well understood. In this study, we examined the effects of static and cyclic uniaxial stretch on macrophage inflammatory and healing activation. We found that cyclic stretch altered macrophage morphology and responses to IFNγ/LPS and IL4/IL13. Interestingly, we found that both static and cyclic stretch suppressed IFNγ/LPS induced inflammation. In contrast, IL4/IL13 mediated healing responses were suppressed with cyclic but enhanced with static stretch conditions. Mechanistically, both static and cyclic stretch increased expression of the integrin CD11b (α_M_ integrin), decreased expression of the mechanosensitive ion channel Piezo1, and knock down of either CD11b or Piezo1 through siRNA abrogated stretch-mediated changes in inflammatory responses. Moreover, we found that knock down of CD11b enhanced the expression of Piezo1, and conversely knock down of Piezo1 enhanced CD11b expression, suggesting the potential for crosstalk between integrins and ion channels. Finally, stretch-mediated differences in macrophage activation were also dependent on actin, since pharmacological inhibition of actin polymerization abrogated the changes in activation with stretch. Together, this study demonstrates that the physical environment synergizes with biochemical cues to regulate macrophage morphology and function, and suggests a role for CD11b and Piezo1 crosstalk in mechanotransduction in macrophages.

## Introduction

Mechanical cues are present in tissues throughout the body, and studies over the last several decades have shown that these signals play a major role in influencing cellular form and function (1, 2). Active mechanical forces including stretch, shear stress, compression, and tension are now widely appreciated to be essential for the function of cells within tissues that regularly endure stress and strain, such as cardiovascular or musculoskeletal tissues (3–7). In contrast, much less is known about how these forces influence immune cells, despite the fact that immune cells exist, function, and migrate throughout the body, including within mechanically active tissues (8). Macrophages are innate immune cells that naturally reside within tissues, or are recruited from blood monocytes to tissues during injury or infection. These cells play a critical role in pathogen or damage surveillance and promoting inflammation and wound healing. This diversity in macrophage function stems from their ability to respond dynamically to cues in their microenvironment (9–11). While the effects of soluble or biochemical stimuli, including pathogens- or damage-associated molecular patterns, cytokines, and chemokines on macrophage function are well- characterized, the role of physical stimuli in regulating macrophage function is not as well understood.

Several recent studies have found that physical cues indeed influence macrophage function. For example, macrophages respond to physical features of biomaterials such as surface grooves or micropatterned adhesive lines and elongate, aligning in the direction of grooves or lines, and polarize towards an alternatively activated phenotype (12, 13). In addition, increasing the stiffness of substrates upon which macrophages are cultured enhances their response to inflammatory or wound healing stimuli, suggesting that mechanical cues have the ability to tune the macrophage response to soluble agonists (14, 15). In response to varying degrees of mechanical stretch, macrophages have been reported to change their morphology, enzymatic activity, proliferation, and activation states (16–19). For example, cyclic stretch was observed to suppress the expression of the inflammatory cytokine IL1β (18). However, the molecular mediators responsible for macrophage mechanotransduction remain elusive.

Mechanoreceptors on the cell surface including integrins and stretch-activated ion channels are major transducers of external physical cues, leading to changes in biochemical signals within the cell (20, 21). Integrins, or adhesion receptors that bind to the extracellular matrix (ECM), cluster in response to force, directly transmit mechanical signals through their connection with the cytoskeleton, and contribute to the activation of intracellular signal transduction pathways (22). In macrophages, integrins are essential in the modulation of various cell functions, such as motility, phagocytosis, and activation, and are also thought to play a role in mechanosensation (23–26). Stretch-activated ion channels, such as Piezo1, respond to changes in membrane tension and transduce external physical stimuli into electrochemical activity, also influencing signaling and cell behavior (20, 21, 27). Moreover, Piezo1 activity is thought to enhance integrin activation and regulate actin polymerization (28–32). A few recent studies including our own work have identified a role for Piezo1 in mechanosensation of hydrostatic pressure, fluid shear stress, and environmental stiffness in myeloid cells (28, 33, 34). Piezo1 activity was also found to promote inflammation (28, 33, 35). However, the role of integrins, Piezo1, and the interplay between these molecules in macrophages, specifically in the context of stretch mechanotransduction is yet to be studied.

In this study, we investigated the effects of cyclic uniaxial stretch on macrophage behavior. We subjected murine bone marrow derived macrophages (BMDMs) to IFNγ/LPS (pro-inflammatory, referred to as M1) or IL4/IL13 (pro-healing, referred to as M2) stimuli along with a 5%, 10%, or 20% cyclic or static uniaxial strain. We observed that uniaxial cyclic stretch led to elongation and alignment of macrophages in the direction of stretch, with a small percentage of cells aligning perpendicularly. While cyclic stretch alone had no influence on macrophage activation, stretch in conjunction with soluble stimuli altered the expression of inflammatory or healing responses. Mechanistically, we found that CD11b (integrin α_M_) expression was enhanced and Piezo1 expression diminished with stretch, and siRNA-mediated knockdown of either receptor led to abrogation of stretch-mediated effects. Moreover, we also observed the potential for crosstalk between CD11b and the mechanically activated ion channel Piezo1. We found that suppressing the expression of one molecule (CD11b/Piezo1) resulted in increased expression of the other molecule. Functionally, reduction in CD11b was observed to enhance Piezo1 expression and resulted in increased inflammatory activation regardless of the presence of stretch. Reduced Piezo1 expression, on the other hand, enhanced CD11b expression and suppressed inflammation independent of stretch. Finally, we found that actin is involved in the transduction of mechanical stretch as pharmacological inhibition of actin polymerization prevented stretch-mediated changes in macrophage activation. Together, these findings suggest that soluble and physical stimuli synergize to alter macrophage function and point to a pivotal role of CD11b and Piezo1 in macrophage sensing of mechanical stretch.

## Materials and Methods

### Cell Isolation and Culture

BMDMs were obtained from the femurs of 6-12 week old female C57BL/6J mice (Jackson Labs). Cells were collected by flushing the bone marrow of the femur with DMEM supplemented with 10% heat-inactivated FBS, 2mM l-glutamine, 1% penicillin/streptomyocin (all from Thermo Fisher), and a 10% conditioned media produced from CMG 14-12 cells expressing recombinant murine macrophage colony stimulating factor (MCSF), which induces differentiation of bone marrow cells to macrophages. To remove red blood cells, the collected bone marrow cells were treated with a red cell lysis buffer, and then centrifuged before being resuspended in the previously mentioned media. After 7 days, the cells were harvested using an enzyme-free dissociation buffer (Fisher Scientific) and seeded onto stretchable silicone-based experimental substrates coated with fibronectin using a 10µg/mL solution. BMDMs were seeded onto the experimental substrates at a density of 2 x 10^5^ cells per substrate (~3.9 x 10^4^ cells/cm^2^). All experiments involving murine macrophages were performed in compliance with the University of California, Irvine’s Institutional Animal Care and Use Committee.

### Application of Static and Cyclic Stretch

The fabrication and validation of the uniaxial cell stretching device used in this study have been previously described (36). Once seeded on the experimental substrates, the cells were incubated for 24 hours at 37°C prior to treatment with soluble stimuli and application of stretch. Following incubation, soluble agonists were added to the wells resulting in either unstimulated, 0.3ng/mL IFNγ (R&D Biosystems) and LPS (Sigma), or 0.1ng/mL IL4 and IL13 (both from BioLegend) containing media. Once the media was replenished static and cyclic uniaxial stretch at a 5%, 10%, or 20% stretch amplitude was initiated for a period of 18 hours. For experiments involving the modulation of adhesion, BMDMs were allowed to adhere for 4 hours prior to stimulation and the application of stretch. In addition, for experiments involving the reduction of CD11b or Piezo1 expression, unstimulated macrophages were exposed to non-target, CD11b, or Piezo1 siRNA (all Dharmacon) in a Nucleofector^®^ solution obtained from a primary cell 4D-Nucleofector^®^ kit (Lonza). The cell suspension was loaded into Nucleocuvettes^®^ and transfection was accomplished through the use of a 4D-Nucleofector^®^ system (Lonza). Following transfection, the cells were supplemented with warm media before being seeded onto experimental substrates. The transfected cells were allowed to adhere for 24-72 hours prior to stimulation and stretch for an additional 18 hours.

### Western Blotting

Following the application of stretch, the cells were rinsed with PBS before being exposed to a lysis buffer, a combination of RIPA lysis buffer and 1% protease inhibitor (both from Fisher Scientific). The substrates were scraped to release the adhered cells and the lysate was collected. The lysate was spun at 14000rpm for 15 minutes and the supernatant was obtained. The proteins were denatured through the use of a Laemmli buffer supplemented with 5% 2-mercaptoethanol at 95°C for 10 minutes before each sample was loaded into a well of a 4-15% mini-PROTEAN™ precast gel (all from Biorad). Gel electrophoresis resulted in the separation of proteins before being transferred onto nitrocellulose membranes through the use of the iBlot dry blotting system (Thermo Fisher Scientific). Following electroblotting, the membranes were blocked using 5% nonfat milk in TBST overnight at 4°C. After 30 minutes of washing in TBST, the membranes were probed with one of the following primary antibodies for 1 hour at room temperature: rabbit anti-iNOS (Abcam), goat anti-arginase-1 (Abcam), rabbit anti-CD11b (Abcam), or mouse anti-GAPDH (BioLegend), used as a loading control. An additional 30 minutes of washing in TBST followed before the membranes were probed with secondary antibodies at room temperature for 1 hour. The membrane was then washed in TBST and immersed into a chemiluminescent HRP substrate solution (Thermo Scientific) and imaged using a ChemiDoc XRS System (Biorad).

### Analysis of Cell Viability

Following cyclic stretch, cells were collected and frozen at -80°C, and analysis of cell viability was conducted using the Cyquant cell proliferation assay kit (Fisher Scientific). The assay was conducted following the manufacturer’s instructions.

### ELISA

Following cyclic stretch, the supernatants were collected and analyzed for the presence of TNF-α, IL-6, and MCP-1 using ELISA kits (BioLegend). The assays were conducted following the manufacturer’s instructions.

### Immunofluorescence

BMDMs isolated from WT mice were used for actin and CD11b staining, and BMDMs isolated from *Piezo1^P1-tdT^* mice, which express a Piezo1-tdTomato fusion protein that is used to label endogenous Piezo1 channels, were used for visualizing Piezo1 (34, 37). For actin staining, BMDMs were fixed in 4% paraformaldehyde following the application of stretch or siRNA knockdown. The fixed cells were washed in PBS prior to permeabilization in 0.1% Triton-X. Following additional PBS washes the cells were incubated with Alexa Fluor 488 phalloidin (Fisher Scientific), diluted 1:100 in PBS, and Hoechst (Invitrogen), diluted 1:2000 in PBS, for 30 minutes at room temperature. The cells were further rinsed in PBS prior to being mounted onto glass slide. For CD11b or Piezo1 staining, the cells were blocked in 2% BSA following fixation prior to being incubated with rat anti-CD11b (BioLegend) and rabbit anti-RFP (Rockland) primary antibodies, diluted 1:50 for CD11b and 1:400 for RFP antibodies in 2% BSA for 1 hour at room temperature. The cells were then repeatedly washed with 2% BSA and incubated with donkey anti-rat (Jackson Immunoresearch Laboratories Inc) or goat anti-rabbit (Thermo Fisher Scientific) secondary antibodies, diluted 1:200 or 1:800 in 2% BSA, for 1 hour at room temperature, respectively. After repeated washing with 2% BSA, the cells were incubated with Alexa Fluor 488 phalloidin (Fisher Scientific), diluted 1:100 in PBS, and Hoechst (Invitrogen), diluted 1:2000 in PBS, for 30 minutes at room temperature. The cells were thoroughly washed with PBS, before being mounted onto a glass slide and imaged using a Zeiss LSM780 confocal microscope.

### RNA Isolation and Quantitative Real-Time PCR

Cells were lysed using TRI Reagent (Sigma-Aldrich) and RNA was isolated using manufacturer's protocols. cDNA was generated using a High-Capacity cDNA Reverse Transcription Kit (Applied Biosystems) and qRT-PCR was performed using PerfeCTa® SYBR® Green SuperMix (QuantaBio). All assays were performed using manufacturer's protocols. The primers used in this study include: *Arg1* (forward, CTCTGTCTTTTAGGGTTACGG and reverse, CTCGAGGCTGTCCTTTTGAG), *Mrc1* (forward, TGTTTTGGTTGGGACTGACC and reverse, TGCAGTAACTGGTGGATTGTC), *Retnla* (forward, GCCAATCCAGCTAACTATCCC and reverse, AGTCAACGAGTAAGCACAGG), *Chi3l3* (forward, AGTGCTGATCTCAATGTGGATTC and reverse, TAGGGGCACCAATTCCAGTC), *Itgam* (forward, ATGGACGCTGATGGCAATACC and reverse, TCCCCATTCACGTCTCCCA), *Itgb1* (forward, ATGCCAAATCTTGCGGAGAAT and reverse, TTTGCTGCGATTGGTGACATT), *Itgb2* (forward, TCACCTTCCAGGTAAAGGTCAT and reverse, AGTTTTTCCCAATGTAGCCAGA), *Itgb3* (forward, CCCCGATGTAACCTGAAGGAG and reverse, GAAGGGCAATCCTCTGAGGG), and *Piezo1* (forward, GTTACCCCCTGGGAACATCT and reverse, TTCAGGAGAGAGGTGGCTGT).

### Image Analysis

Phase contrast images were captured following 18 hours of cyclic mechanical stretch using the EVOS cell imaging system (Thermo Fisher Scientific). At least 150 cells from each condition, per experiment, were manually outlined and analyzed using ImageJ, which fits an ellipse to each outlined cell. The ratio of the major and minor axis of the fitted ellipse were used to determine the aspect ratio. The software was also used to compute the angle of the major axis relative to the direction of stretch and the area of each cell. Cells aligned in the direction of the stretch will, therefore, have an angle of 0°. To quantify the percent of aligned cells, alignment parallel to the uniaxial strain was defined as having an angle between -30° to 30° and alignment perpendicular to the strain was defined as an angle between -60° to -90° and 60° to 90°. Percentages were then obtained by taking the ratio of aligned cells to the total number of cells circled. Quantification of mean fluorescence intensity was performed similarly. Approximately 50 cells in each condition were outlined per experiment and the mean intensity was computed for each cell using ImageJ.

### Flow Cytometry

Unstimulated macrophages were gently scraped from the surface of the experimental substrates and fixed with 4% paraformaldehyde. After repeated washing with PBS, the cells were resuspended in 1% BSA and incubated at 4°C overnight before being blocked with anti CD16/32 antibodies (Tonba) for 45 minutes at room temperature. The cells were then incubated for an additional 45 minutes at room temperature with PE anti-mouse/human CD11b antibodies (clone: M1/70) or PE rat IgG2b antibodies (clone: RTK4530), used as an isotype control (both from BioLegend). Following repeated washes, flow cytometry was performed using BD LSRII (BD Bioscience) and quantification of the median fluorescent intensity of the obtained data was performed using FlowJo software (FlowJo, LLC).

### Statistical Analysis

Data are presented as the mean ± standard deviation of the mean across at least three independent experiments, unless otherwise noted. Comparisons were performed using a two-tailed Student’s t-test or paired t-test and *p* < 0.05 was considered significant.

## Results

### Biochemical Stimuli and Mechanical Stretch Synergize to Regulate Macrophage Morphology and Function

**Figure 1:**
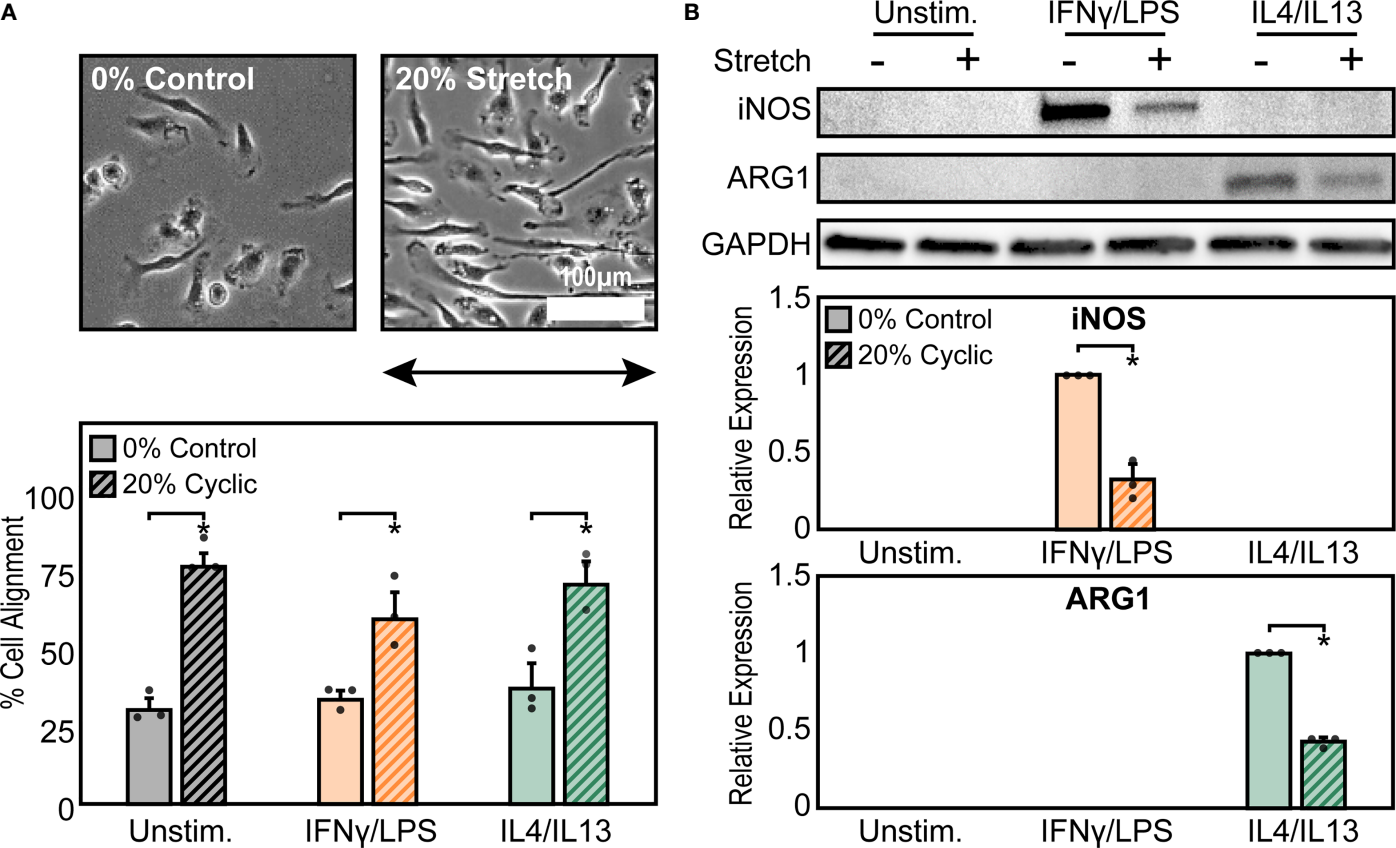
Mechanical stretch alters macrophage morphology and activation. **(A)** Phase contrast images (top) and quantification of percent cell alignment (bottom) of unstimulated macrophages exposed to 0% and 20% cyclic uniaxial stretch. Uniaxial strain was applied in the horizontal direction as indicated by the arrow.**(B)** Representative western blots (top) and corresponding quantification for iNOS (middle) and ARG1 (bottom) for unstimulated, IFNγ/LPS, and IL4/IL13 stimulated macrophages. Values were normalized to GAPDH and made relative to IFNγ/LPS or IL4/IL13 stimulated and 0% stretch conditions, respectively. Error bars indicate standard deviation of the mean for three separate experiments and *p < 0.05 when compared to the indicated condition as determined by Student’s t-test **(A)** or paired t-test **(B)**.

To explore the effects of cyclic mechanical strain and soluble stimuli in regulating macrophage form and function, a uniaxial cell stretcher was used to mechanically stretch unstimulated (Unstim.), IFNγ/LPS, or IL4/IL13 stimulated BMDMs. Cells were cultured on stretchable substrates overnight prior to stimulation and exposure to a 1 Hz, 20% uniaxial strain for 18 hrs, similar stretch amplitude to what has previously been used to replicate mechanical stretch experienced within the heart (38). Given our own work identifying the importance of cell shape in regulating macrophage function (12), we first analyzed the role of cyclic forces in modulating macrophage cell morphology when compared to static controls (**Figure 1A**). Unstimulated macrophages exhibited a range of aspect ratios and had no distinct orientation. Macrophages stimulated with IFNγ/LPS, adopted a flat and round cell shape, whereas macrophages stimulated with IL4/IL13, were elongated, as we have previously observed in macrophages cultured on other material surfaces (14). When exposed to cyclic uniaxial strain, macrophages in all soluble stimulation conditions display alignment parallel to the uniaxial stretch (**Figure 1A**). Interestingly, stretch also caused perpendicular alignment of a small fraction of cells, although to a lesser extent for the unstimulated and IL4/IL13 stimulated macrophages when compared to an unstretched control (**Figure S1A**). This could be attributed to over-extending of the stretchable membranes resulting in the generation of perpendicular strains, as has previously been reported (36). Increases in elongation, or the ratio of the length of the major axis to the length of the minor axis, were observed only in the IFNγ/LPS stimulated macrophages exposed to cyclic strain (**Figure S1B**), suggesting that stretch causes classically activated macrophages to deviate from their typical round morphology and instead adopt a more elongated morphology. Cell area across all stimulation and stretch conditions were unchanged (**Figure S1C**). Together, these results show that cyclic uniaxial stretch leads to changes in macrophage elongation, particularly in the IFNγ/LPS-stimulated condition, and alignment along the direction of stretch.

Given that we have previously found that cell elongation is associated with enhanced wound healing and diminished inflammatory responses (12, 39), we next investigated the role of cyclic mechanical stretch in influencing macrophage function. Following stretch, we observed no changes in the expression of the inflammatory marker iNOS or the healing marker arginase-1 (ARG1) in stretched unstimulated macrophages compared to unstretched controls (**Figure 1B**). This finding suggests that stretch, and its resulting effects on cell morphology, alone are unable to regulate macrophage activation, in contrast to what we have previously found with elongation induced healing activation in BMDMs cultured on micropatterned line or grooves (12, 13). However, the addition of IFNγ/LPS enhanced expression of iNOS, as expected, and expression significantly decreased upon application of stretch. Similarly, ARG1 expression in IL4/IL13 stimulated macrophages decreased with stretch (**Figure 1B**). Moreover, no significant differences in macrophage viability were observed with stretch, thus confirming that stretch was able to influence IFNγ/LPS or IL4/IL13 induced inflammatory and healing responses, respectively (**Figure S2**).

**Figure 2:**
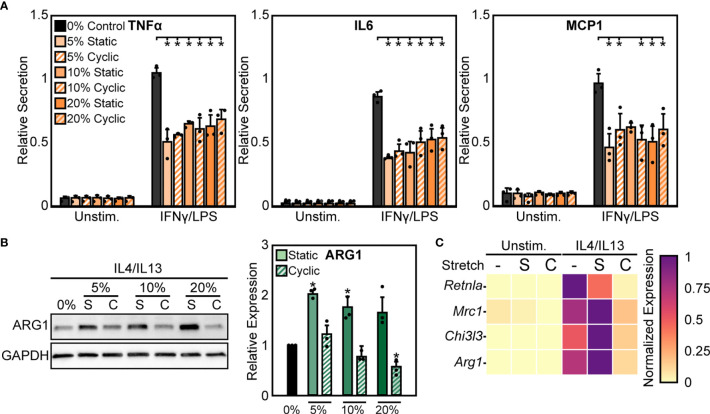
Static and cyclic stretch differentially regulate macrophage inflammatory and healing activation. **(A)** Secretion of TNFα, IL6, and MCP1 for unstimulated and IFNγ/LPS stimulated macrophages exposed to 0%, 10%, and 20% static and cyclic strains. Values are normalized to a 0% stretch and IFNγ/LPS stimulated internal control within each biological replicate. **(B)** Representative western blots (left) and quantification (right) of ARG1 expression for three independent experiments in IL4/IL13 stimulated macrophages exposed to 0%, 10%, and 20% static and cyclic stretch conditions for 18 hrs. Values normalized and statistical comparisons made to IL4/IL13 stimulated condition. **(C)** Gene expression of *Arg1*, *Chi3l3*, *Mrc1*, and *Retnla* for unstimulated and IL4/IL13 stimulated macrophages exposed to 0% (-), 20% static (S), and 20% cyclic (C) strains. Data represents three independent experiments and made relative to the highest expressing condition within each gene. Error bars indicate standard deviation of the mean for three separate experiments and *p < 0.05 when compared to the indicated condition as determined by Student’s t-test **(A)** or paired t-test with Bonferroni correction used for multiple comparisons **(B)**.

We next sought to further characterize functional differences in macrophage responses to mechanical strain through exposing BMDMs to both cyclic and static stretch at 5%, 10%, and 20% amplitudes. Similar to our previous results, unstimulated macrophages displayed no significant differences in inflammatory marker secretion with stretch alone, but in the presence of IFNγ/LPS stimulation cells exhibited a significant decrease in the secretion of inflammatory markers TNFα, IL6, and MCP1 in response to static and cyclic stretch regardless of strain amplitude, when compared to the unstretched and IFNγ/LPS stimulated control conditions (**Figure 2A**). In contrast, IL4/IL13 stimulated BMDMs displayed differential expression of healing markers with stretch. More specifically, static stretch increased the expression of ARG1 regardless of stretch amplitude, whereas increasing amplitudes of cyclic stretch resulted in lower ARG1 expression, with significant decreases observed at both 10% and 20% amplitudes (**Figure 2B**). Stretch had similar effects on additional healing markers, with increases in expression resulting from 20% static stretch and decreases observed with a 20% cyclic stretch, as measured by qRT-PCR (**Figure 2C**). These data suggest that soluble stimuli and stretch act synergistically to modulate the function of macrophages, and that inflammatory activation is inhibited consistently by different stretch regimes, whereas wound healing responses are more varied depending on the stretch profile.

### Increased CD11b Expression Dampens Stretch-Mediated Changes in Inflammatory Activation

**Figure 3:**
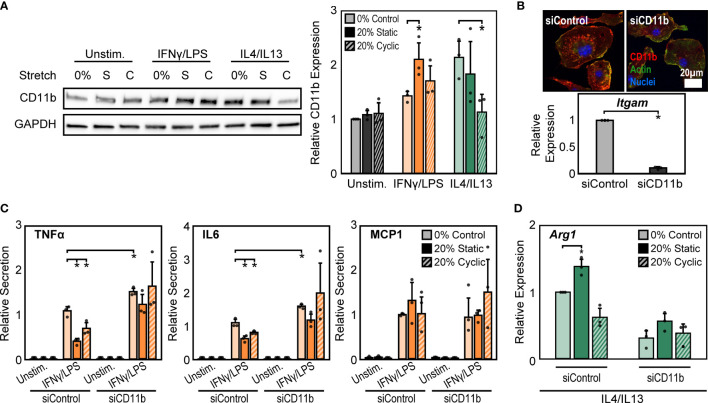
CD11b is required for stretch-mediated changes in macrophage activation. **(A)** Representative Western blot of CD11b and GAPDH for unstimulated, IFNγ/LPS, and IL4/IL13 stimulated macrophages exposed to 0% and 20% static and cyclic stretch (left). Quantification of average across three independent experiments for CD11b expression (right). Values were normalized to GAPDH and made relative to 0% stretch and unstimulated condition. **(B)** Representative immunofluorescence images (top) and quantification of relative *Itgam* gene expression in unstimulated macrophages treated with non-target (siControl) or CD11b (siCD11b) siRNA. Data relative to siControl condition. **(C)** Secretion of TNFα, IL6, and MCP1 for unstimulated and IFNγ/LPS stimulated macrophages treated with siControl or siCD11b and exposed to either 0% control, 20% static, or 20% cyclic stretch. Data normalized to a siControl and IFNγ/LPS treated internal control exposed to 0% stretch within each biological replicate. **(D)** Relative *Arg1* gene expression in IL4/IL13 stimulated and siControl or siCD11b treated BMDMs exposed to 0% control, 20% static, or 20% cyclic stretch. Data relative to 0% siControl condition. Error bars indicate standard deviation of the mean for three separate experiments and *p < 0.05 when compared to the corresponding 0% stretch condition as determined by Student’s t-test **(A, C)** or paired t-test **(B, D)**.

Macrophage interactions with matrix-coated surfaces and the subsequent remodeling of the cytoskeleton are facilitated by adhesion molecules including integrins, which are thought to be critical transducers of physical stimuli including stretch (21). Among the many integrin subtypes, CD11b, or α_M_ integrin, is the most abundant integrin in macrophages and its expression is often used as a marker of macrophage differentiation (40). We measured the expression of CD11b under the different conditions described above and found that 20% static or 20% cyclic stretch alone led to no differences in CD11b expression in unstimulated macrophages (**Figure 3A**). However, IFNγ/LPS stimulation caused significant increases in CD11b expression, consistent with our earlier work (24), and further increases were observed following static or cyclic stretch **(** **Figure 3A** **)**. IL4/IL13 stimulation also increased CD11b expression. Stretch, on the other hand, decreased IL4/IL13-induced CD11b expression with significant decreases observed following 20% cyclic stretch (**Figure 3A**).

CD11b has previously been shown to play an important role in regulating macrophage inflammatory responses. Macrophages from CD11b deficient mice exhibit enhanced inflammation in response to LPS, suggesting that CD11b negatively regulates macrophage inflammatory activation (26). However, the role of this integrin in transducing mechanical stretch in macrophages has not been explored. To better understand the role of CD11b in stretch mechanotransduction, we first investigated changes in macrophage activation following exposure to CD11b siRNA (siCD11b). We found that siCD11b treated cells had reduced integrin expression when compared to non-target (siControl) treated controls, confirming knock down of CD11b (**Figure 3B**). In addition, siCD11b treated cells had enhanced secretion of inflammatory cytokines, particularly TNFα and IL6, when compared to siControl treated cells, consistent with previous work using CD11b knockout macrophages (26). Furthermore, knock down of CD11b abrogated stretch-induced inhibition of inflammation, which was observed in siControl treated macrophages (**Figure 3C**). Instead, the inflammatory response to IFNγ/LPS and stretch was moderately enhanced. In contrast, IL4/IL13 and siCD11b treatment reduced the expression of *Arg1* and prevented any stretch induced changes, suggesting that CD11b also plays an important role in the effects of stretch on healing responses (**Figure 3D**).

**Figure 4:**
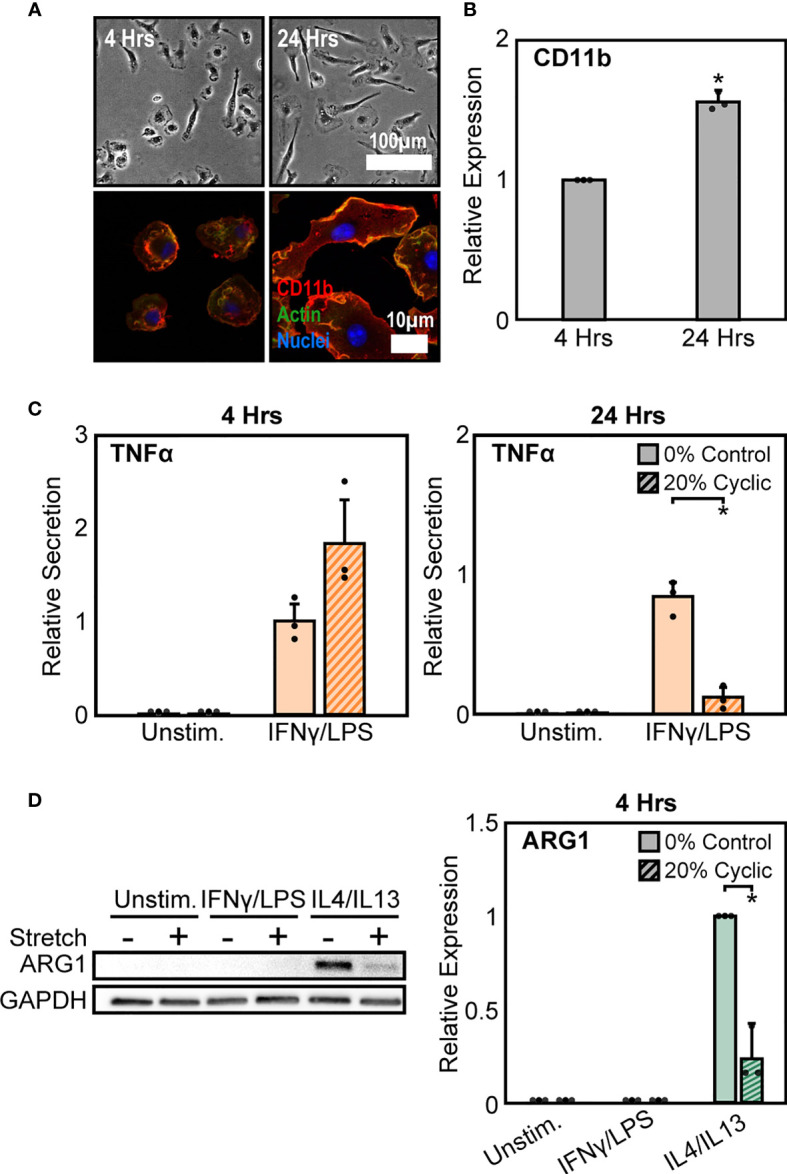
Modulation of CD11b by adhesion time regulates stretch-mediated macrophage inflammatory responses. **(A)** Phase contrast images (top) of macrophages following 4 hrs (left) and 24 hrs (right) of adhesion prior to stimulation and stretch. Fluorescence images (bottom) of macrophages labelled for CD11b (red), actin (green), and nuclei (blue) following 4 hrs (left) and 24 hrs (right) of culture. **(B)** Averaged relative median fluorescence intensity across three independent experiments of CD11b as measured by flow cytometry. Values normalized to 4 hrs adhesion condition. **(C)** Secretion of TNFα for unstimulated and IFNγ/LPS stimulated macrophages exposed to 0% and 20% cyclic stretch after 4 hrs (left) and 24 hrs (right) of adhesion. Values are normalized to a 0% stretch IFNγ/LPS internal control within each biological replicate. **(D)** Representative Western blots (left) and corresponding quantification for ARG1 (right) for unstimulated, IFNγ/LPS, and IL4/IL13 stimulated macrophages allowed to adhere for 4 hrs prior to stimulation and stretch. Values were normalized to GAPDH and made relative to IL4/IL13 stimulated and 0% stretch conditions, respectively. Error bars indicate standard deviation of the mean for three separate experiments and * *p* < 0.05 when compared to the corresponding 0% stretch condition as determined by Student’s t-test **(C)** or paired t-test **(B, D)**.

As a second method to modulate CD11b expression, we altered the time of adhesion to the substrate prior to stretch. We found that expression of CD11b was dependent on time of adhesion to the substrate, with significantly higher CD11b expressed at longer adhesion times (**Figure 4A**). After 4 hrs of adhesion, macrophage cell spreading was heterogeneous, with some cells clearly adhered and spread whereas others just adhering and beginning to spread. In contrast, after 24 hrs of adhesion, cells appeared to be more homogenous and well-spread (**Figure 4B**). Interestingly, similar changes in cell alignment and morphology were observed following stretch in cells adhered for 4 hrs as was previously noted for cells adhered for 24 hrs (**Figure S3** **)**. However, while morphology was similar, the stretch mediated inflammatory responses differed as reduced adhesion times prevented strain induced negative regulation of inflammation, and in fact caused higher levels of TNFα compared to unstretched controls (**Figure 4C**). No such changes were observed with respect to healing responses, as both 4 hrs (**Figure 4D**) and 24 hrs of adhesion (**Figure 1B** **)** resulted in suppressed ARG1 expression. Together, these data suggest that IFNγ/LPS and stretch treatment enhances CD11b expression, which reduces the inflammatory response, while IL4/IL13 and cyclic stretch treatment suppresses CD11b expression resulting in a decreased healing response.

### Integrin and Ion Channel Crosstalk Could Regulate Macrophage Response to Stretch

**Figure 5:**
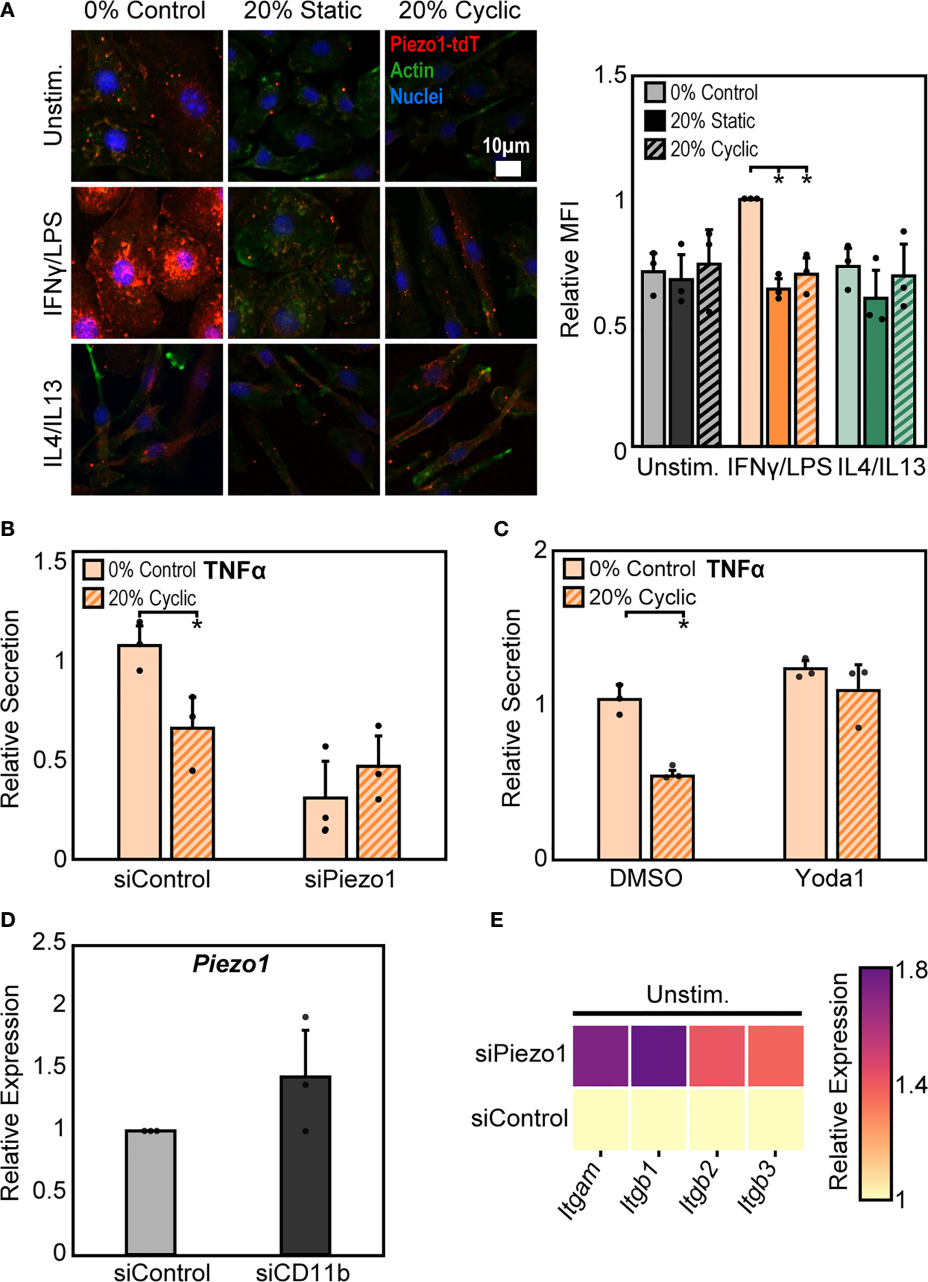
Crosstalk between Piezo1 and CD11b mediates macrophage response to stretch. **(A)** Representative immunofluorescence images (left) and quantification of mean Piezo1-tdT intensity in unstimulated, IFNγ/LPS, and IL4/IL13 treated macrophages exposed to 0% control, 20% static, or 20% cyclic stretch. Data normalized to the 0% control condition. **(B)** Secretion of TNFα in IFNγ/LPS stimulated macrophages treated with siControl or siPiezo1 and exposed to either 0% or 20% cyclic stretch. Data normalized to a siControl treated internal control exposed to 0% stretch within each biological replicate. **(C)** Secretion of TNFα in IFNγ/LPS stimulated macrophages treated with DMSO or Yoda1 and exposed to either 0% or 20% cyclic stretch. Data normalized to a DMSO treated internal control exposed to 0% stretch within each biological replicate. **(D)** Relative *Piezo1* gene expression in unstimulated macrophages treated with non-target (siControl) or CD11b (siCD11b) siRNA. Gene expression is normalized to the siControl treated condition. **(E)** Relative *Itgam, Itgb1, Itgb2*, and *Itgb3* gene expression in unstimulated and siControl or siPiezo1 treated macrophages. Gene expression is normalized to the siControl treated condition. Error bars indicate standard deviation of the mean for three separate experiments and *p < 0.05 when compared to the corresponding 0% stretch condition as determined by Student’s t-test.

In addition to integrins, mechanically activated ion channels are also present on the cell surface and are involved in transducing mechanical stimuli and modulating cell function. Influx of Ca^2+^ through ion channels has been shown to play important roles in regulating macrophage activation and adhesion (28, 41, 42). Of these channels, Piezo1 has recently been shown to be highly expressed in macrophages and is known to sense and transduce cyclic hydrostatic pressure, shear stresses, and stiffness, while also regulating macrophage inflammatory activation (28, 33–35). To evaluate the role of Piezo1 in regulating stretch-mediated macrophage inflammatory responses we examined Piezo1 expression, and modulated Piezo1 activity or expression using pharmacologic and genetic approaches. We first evaluated changes in Piezo1 expression following stretch using cells from a mouse expressing tdTomato fused to endogenously expressed Piezo1. We visualized tdTomato signal after stretch using fluorescence microscopy, and found that IFNγ/LPS treatment resulted in enhanced Piezo1 channel expression, as has previously been observed (34). In addition, both static and cyclic stretch suppressed IFNγ/LPS mediated channel expression (**Figure 5A**). No such differences were observed in the Unstim. or IL4/IL13 conditions. To determine the significance of reduced Piezo1 expression in sensing stretch, we next treated BMDMs with Piezo1 siRNA (siPiezo1) prior to IFNγ/LPS stimulation and stretch. We found that siPiezo1 treated BMDMs had reduced inflammatory responses regardless of stretch when compared to the siControl condition (**Figure 5B**). Furthermore, we also evaluated changes in stretch-induced macrophage inflammation following Piezo1 activation. We treated BMDMs with 5 µM Yoda1, a Piezo1 specific agonist (43), prior to stimulation with IFNγ/LPS and stretch, and found that Yoda1 treatment enhanced TNFα secretion in both control and stretch conditions and prevented stretch-mediated decreases in cytokine production as was observed in DMSO controls (**Figure 5C**). These data suggest that mechanical stretch downregulates Piezo1 expression, thus suppressing IFNγ/LPS mediated inflammation, and the addition of Yoda1 rescues Piezo1 activity which, in turn, enhances inflammation.

Our findings show that stretch increases CD11b expression while concomitantly decreasing Piezo1 expression, each of which lead to decreases in inflammation in response to IFNγ/LPS. Piezo1 has been shown to enhance the activation of integrins in different cell types (28–30), so we next explored a potential connection between CD11b and Piezo1 in our system. Interestingly, we found that cells treated with siCD11b had increased Piezo1 expression compared to siControl treated cells (**Figure 5D**). In contrast, cells treated with siPiezo1 had increased expression of *Itgam, Itgb1, Itgb2, and Itgb3* (integrin α_M_, β_1_, β_2_, β_3,_ respectively) (**Figure 5E**). These data suggest a potential interplay between integrins and ion channels, where the expression of one leads to the downregulation of the other. In addition, high CD11b and low Piezo1 are associated with a reduced inflammatory response to IFNγ/LPS and low CD11b and high Piezo1 are associated with higher inflammation. Finally, stretch-induced changes in expression of these surface proteins may be responsible for the changes in inflammation associated with stretched conditions.

### Stretch-Mediated Modulation of Actin Regulates Macrophage Activation

**Figure 6:**
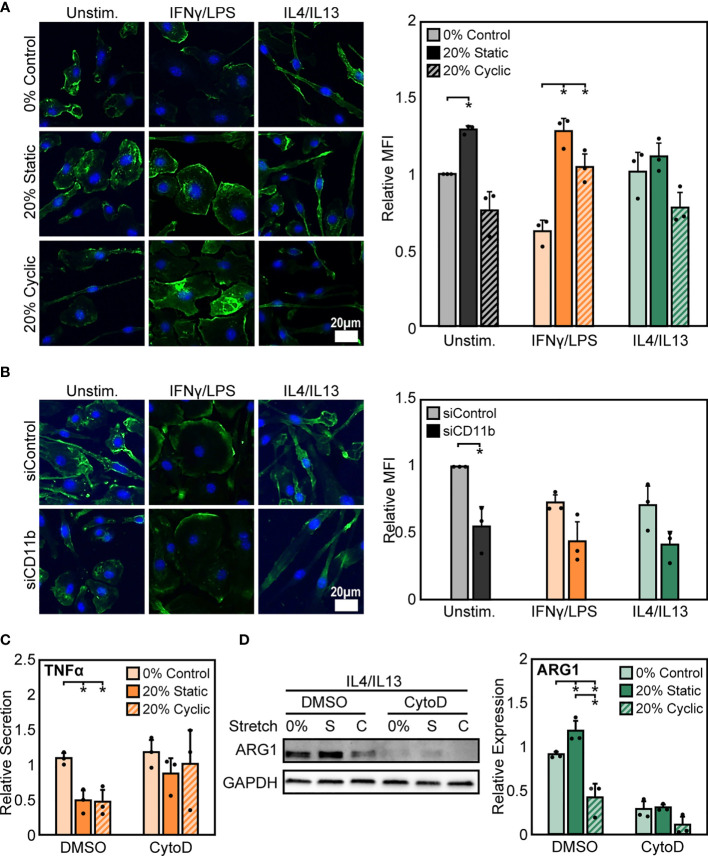
Stretch-induced changes in macrophage activation require modulation of actin. **(A)** Representative images of F-actin in unstimulated, IFNγ/LPS, and IL4/IL13 stimulated macrophages exposed to 0%, 20% static, and 20% cyclic stretch. Quantification of mean F-actin fluorescence intensity across three independent experiments (right). Data normalized to the unstimulated and 0% stretch control. **(B)** Representative images of F-actin in unstimulated, IFNγ/LPS, and IL4/IL13 stimulated macrophages exposed to siControl or CD11b siRNA. Quantification of mean F-actin fluorescence intensity across three independent experiments (right). Values normalized to unstimulated and siControl condition. **(C)** Secretion of TNFα in IFNγ/LPS stimulated macrophages treated with DMSO or CytoD and exposed to 0% and 20% static or cyclic strains. Values are normalized to a DMSO, 0% stretch, and IFNγ/LPS stimulated internal control within each biological replicate. **(D)** Representative Western blot (left) and quantification of ARG1 expression in IL4/IL13 stimulated macrophages treated with DMSO or CytoD and exposed to 0% and 20% static or cyclic strains. Expression is relative to GAPDH. Error bars indicate standard deviation of the mean for three separate experiments and *p < 0.05 when compared to the corresponding 0% stretch condition as determined by paired t-test **(A, B)** and Student’s t-test **(C, D)**.

To probe potential intracellular mediators of stretch, we next investigated the role of actin, a cytoskeletal protein connected to integrins. The cytoskeleton is pivotal to the transduction of various mechanical stimuli, and numerous studies in macrophages and other cell types have shown that inhibition of the cytoskeleton prevents mechanically-mediated changes in cell function (44, 45). To investigate the potential role of actin, we quantified mean fluorescence intensity of phalloidin stained F-actin after stretch and stimulation with IFNγ/LPS or IL4/IL13. We observed that, stimulation alone alters F-actin composition in macrophages, with IFNγ/LPS stimulation resulting in reduced F-actin intensity when compared to unstimulated or IL4/IL13 stimulated macrophages. Following both static and cyclic stretch, we found enhanced F-actin in unstimulated and IFNγ/LPS stimulated cells. In contrast, we observed a modest stretch-induced increase in actin in IL4/IL13 stimulated macrophages (**Figure 6A**). This differential regulation of F-actin composition in IFNγ/LPS stimulated macrophages mirrors the changes observed in stretch-mediated macrophage function, with decreases in actin correlating with decreased levels of inflammation.

To determine the role of CD11b in influencing actin, we measured F-actin intensity in siCD11b and siControl macrophages. We found that siCD11b treatment reduced F-actin intensity when compared to siControl treated cells (**Figure 6B**). Changes in actin due to loss of CD11b could potentially indicate a role for this integrin in establishing cytoskeletal integrity. To further elucidate the role of actin in transducing stretch, we evaluated changes in stretch-induced macrophage activation following exposure to cytochalasin D (CytoD), an actin polymerization inhibitor. We found that CytoD resulted in enhanced IFNγ/LPS induced TNFα secretion with no stretch-mediated reduction in inflammation observed when compared to DMSO controls (**Figure 6C**). In contrast, CytoD treatment suppressed IL4/IL13 induced ARG1 expression in all conditions (**Figure 6D**). Together, our results indicate that the actin cytoskeleton lies downstream of CD11b, and is critical for transducing mechanical stretch.

## Discussion

In this study, we demonstrate that mechanical stretch modulates macrophage morphology and functional response to soluble signals in their microenvironment. In response to cyclic uniaxial stretch, macrophages elongated and aligned in the direction of stretch. The degree of alignment and elongation was dependent on macrophage activation state, where unstimulated and IL4/IL13 stimulated cells displayed significant alignment in the direction of stretch, and IFNγ/LPS stimulated cells displayed significant elongation when stretched, in comparison to a static control. Moreover, a small number of cells were also found to align perpendicularly to the direction of applied stretch, which may be due to perpendicular compressive strains resulting from extension of the silicone substrate. The morphological response of macrophages to strain was consistent with what has been shown previously in other macrophage cell lines (16, 46). Cyclic mechanical stretch not only caused differences in cell morphology, but also resulted in functional changes of macrophages in response to soluble signals. Interestingly, although macrophage elongation has previously been observed to increase healing or alternative activation (ARG1 expression) when cells were cultured on micropatterned lines or grooves (12, 13), elongation caused by cyclic stretch itself did not appear to alter macrophage function. Stretch alone had no significant impact on macrophage inflammatory or healing functions, suggesting that soluble cues are needed in addition to stretch, to synergistically alter macrophage function, as has been observed by others (47). Further characterization of stretch responses showed that static strain promotes IL4/IL13 induced healing activation, whereas high amplitude cyclic stretch suppresses healing activation. Our results are consistent with previous work which shows that static strains enhance healing activation in murine skin (19). In contrast to healing activation, IFNγ/LPS stimulated macrophages were observed to have similar and reduced inflammatory responses when exposed to varying amplitudes of both cyclic and static stretch. While this observation is consistent with some reports, others provide contradictory conclusions as stretch provides no effect or may even enhance inflammatory activation in macrophages (18, 48–52). This, however, could be attributed to the numerous differences in experimental setup (stretch amplitudes, duration, and frequency as well as macrophage source and addition of stimulation relative to onset of stretch), as our studies have shown that differences in the stretch profile and the time of adhesion prior to stretch can have dramatic effects. Nonetheless, our findings indicate that mechanical stimulation modulates macrophage activation in response to soluble stimuli (**Table 1**).

**Table 1:** Summary of stretch mediated changes in macrophage function.

Stimulation	Effects of Stretch	Stretch mediated changes in expression		
		CD11b	Piezo1	Actin
**Unstim.**	No effect	No effect	No effect	Static: enhance
**IFNγ/LPS**	Stretch: suppress inflammation	Stretch: enhance	Stretch: suppress	Stretch: enhance
**IL4/IL13**	Static: enhance healing	Cyclic: suppress	No effect	No effect
	Cyclic: suppress healing			

Changes in stretch mediated effects in macrophage inflammatory/healing responses as well as differences in CD11b, Piezo1, and F-actin expression are summarized. Stretch denotes similar observations between both cyclic and static stretch conditions.

To better understand the molecules responsible for stretch-induced changes in macrophage activation, we analyzed the role of integrins. CD11b is the most highly expressed integrin in macrophages and its expression has been shown to modulate the effects of inflammatory signaling by LPS-induced TLR4 mediated signaling in several myeloid cell types. Using peritoneal macrophages, Han et al. showed CD11b negatively regulates TLR4 since CD11b deficient cells exhibited a higher inflammatory response to LPS when compared to wild type cells (26). We show that stretch-mediated CD11b expression could potentially be responsible for downregulation of LPS-induced TLR4 signaling, since stretch led to higher levels of CD11b and decreases in inflammation with IFNγ/LPS. Cells expressing lower levels of CD11b as induced by siRNA or reduced adhesion time, did not exhibit this response. Moreover, we also show that cyclic stretch mediated reduction in CD11b expression dampens IL4/IL13 induced healing responses. These results suggest that stretch modulates CD11b expression, which impacts the functional response of macrophages to soluble stimuli (**Table 1**). However, future studies will need to further elucidate the effects of stretch on inflammatory and healing signaling pathways.

Mechanically activated ion channels are also present on the cell surface and play a critical role in transducing mechanical stimuli (28, 30, 41, 42). In macrophages, Piezo1 has been shown to play a role in sensing pressure, shear stresses, and stiffness, while also regulating inflammation and healing responses as well as phagocytosis (28, 33, 35, 53). Our data suggest that prolonged exposure to mechanical stretch suppresses Piezo1 expression. We also show that reduction of Piezo1 with siRNA resulted in similarly reduced IFNγ/LPS-mediated inflammatory responses in both control and stretch conditions. In contrast, Yoda1 enhanced inflammatory responses in both control and stretch conditions. Several studies have reported interactions among Piezo1, integrins, and the actin cytoskeleton (28, 30–32). We found that Piezo1 and CD11b are coregulated, with increased expression in CD11b suppressing Piezo1, and vice versa. Furthermore, our data suggest that prolonged mechanical stretch reduces Piezo1-induced inflammation and enhances CD11b-mediated suppression of inflammatory responses. Recent studies have also identified a role for Piezo1 in promoting hypoxia inducible factor alpha (HIF1α) or nuclear factor kappa-light-chain-enhancer of activated B cells (NFκB) activation resulting in enhanced inflammation (33, 34). Given our observations whereby mechanical stretch decreases Piezo1 expression, it is possible that suppressed HIF1α or NFκB could result in dampened inflammation in response to stretch. In contrast to inflammation, our previous work has shown that transient siRNA mediated reduction in Piezo1 expression has no effect in regulating IL4/IL13 mediated healing responses (34). We also found that stretch leads to changes in the actin cytoskeleton (**Table 1**), which is known to influence several macrophage functions including phagocytosis and activation (39, 54, 55). Moreover, inhibition of actin polymerization prevented stretch-mediated changes in macrophage inflammatory and healing responses. Our results indicate a potential role for integrin and ion channel crosstalk to modulate the transduction of stretch through the actin cytoskeleton.

Macrophages *in vivo* reside within tissues, and are also recruited from circulation to tissues throughout the body. Some resident macrophages are continuously exposed to mechanical stresses, whereas recruited macrophages experience dynamic changes in adhesion as they extravasate from the blood vessel into tissue and are exposed to physical stimuli. Inflammatory signals induce immune cell recruitment through upregulation of many adhesion molecules including CD11b (56) or activation of ion channels (28, 33, 35), which likely also affects their perception of mechanical stimuli. However, prolonged exposure to mechanical stress, such as those experienced by lung alveolar macrophages, may desensitize the cells and therefore exhibit limited effects of Piezo1 (33). Our results support this idea since prolonged stretch caused a decrease in Piezo1 expression. Future work examining Piezo1 expression levels and activity of other resident macrophage populations that reside within different mechanical environments will be of significant interest. Our results also show that CD11b and Piezo1 expression levels influence the macrophage inflammatory response during mechanical stretch, and may suggest that stretch differentially impacts macrophages as they progressively adhere within tissues. Differential regulation of macrophages over time may be important for the progression of healing processes.

Our study describes how different mechanical stretch profiles regulate macrophage function, and also provide new insight into the potential role of CD11b, Piezo1, and the actin cytoskeleton in transducing mechanical stimuli. While the current study is limited to the effects of stretch in cells cultured on a two-dimensional substrate, future work will consider macrophages in three-dimensional tissues and subjected to a multitude of mechanical forces, including stretch and fluid shear or interstitial stresses (57). Further studies will be needed to explore the mechanisms by which combinations of mechanical cues affect macrophages in a three-dimensional microenvironment that represent physiological tissues. This work may further our understanding of how mechanical forces contribute to macrophage behavior during homeostasis, wound healing, as well as the progression of inflammatory diseases.

## Data Availability Statement

The raw data supporting the conclusions of this article will be made available by the authors, without undue reservation.

## Ethics Statement

The animal study was reviewed and approved by University of California Institutional Animal Care and Use Committee.

## Author Contributions

HA, VM, CD, MP, and WL designed research. HA, VM, KB, SA, JC, and KJ performed research. HA and WL analyzed data. HA, MP, and WL wrote the paper. All authors contributed to the article and approved the submitted version.

## Funding

This work was supported by the National Institutes of Health (NIH) National institute of Dental and Craniofacial Research (NIDCR) and Office of Director’s Grant DP2DE023319, National Institutes of Allergy and Infectious Diseases (NIAID) R21AI128519 and R01AI151301 (to WL), National Institute for Neurological Disorders and Stroke (NINDS) R01NS109810 (to MP), and the University of California Irvine Undergraduate Research Opportunities Program (UROP). HA was supported by NIH National Institute T32 Training Grant in Cardiovascular Applied Research and Entrepreneurship (5T32 HL116270-3) and American Heart Association Pre-Doctoral Fellowship (20PRE35200220). This study was made possible, in part, through access to the Optical Biology Core Facility of the Developmental Biology Center, a shared resource supported by the Cancer Center Support Grant (CA-62203) and Center for Complex Biological Systems Support Grant (GM-076516) at the University of California, Irvine.

## Conflict of Interest

The authors declare that the research was conducted in the absence of any commercial or financial relationships that could be construed as a potential conflict of interest.

## Publisher’s Note

All claims expressed in this article are solely those of the authors and do not necessarily represent those of their affiliated organizations, or those of the publisher, the editors and the reviewers. Any product that may be evaluated in this article, or claim that may be made by its manufacturer, is not guaranteed or endorsed by the publisher.
