# Supplementary material for: Crosstalk Between CD11b and Piezo1 Mediates Macrophage Responses to Mechanical Cues
Source: Front Immunol. 2021 Sep 22;12:689397. doi: 10.3389/fimmu.2021.689397 (PMC8493066; doi:10.3389/fimmu.2021.689397)
Supplement: Supplementary file 1 [file DataSheet_1.pdf]

Supplementary Material for:

Crosstalk between CD11b and Piezo1 mediates macrophage responses to mechanical cues

Hamza Atcha[1,2], Vijaykumar S. Meli[1,2], Chase T. Davis[1,2] Kyle T. Brumm[1,2], Sara Anis[1,2], Jessica Chin[1,2], Kevin Jiang[1,2], Medha M. Pathak[1,4,5], and Wendy F. Liu[1,2,3,6*]

[1] Department of Biomedical Engineering, University of California, Irvine
[2] The Edwards Lifesciences Center for Advanced Cardiovascular Technology, University of California, Irvine
[3] Department of Chemical Engineering and Materials Science, University of California, Irvine
[4] Department of Physiology and Biophysics, University of California, Irvine
[5] Sue and Bill Gross Stem Cell Research Center, University of California, Irvine
[6] Department of Molecular Biology and Biochemistry, University of California, Irvine

*** Correspondence:**
2412 Engineering Hall
Irvine, CA 92697
Tel: (949) 824-1682
Fax: (949) 824-9968
Email: wendy.liu@uci.edu

* To whom correspondence may be addressed.

1 Supplementary Figures

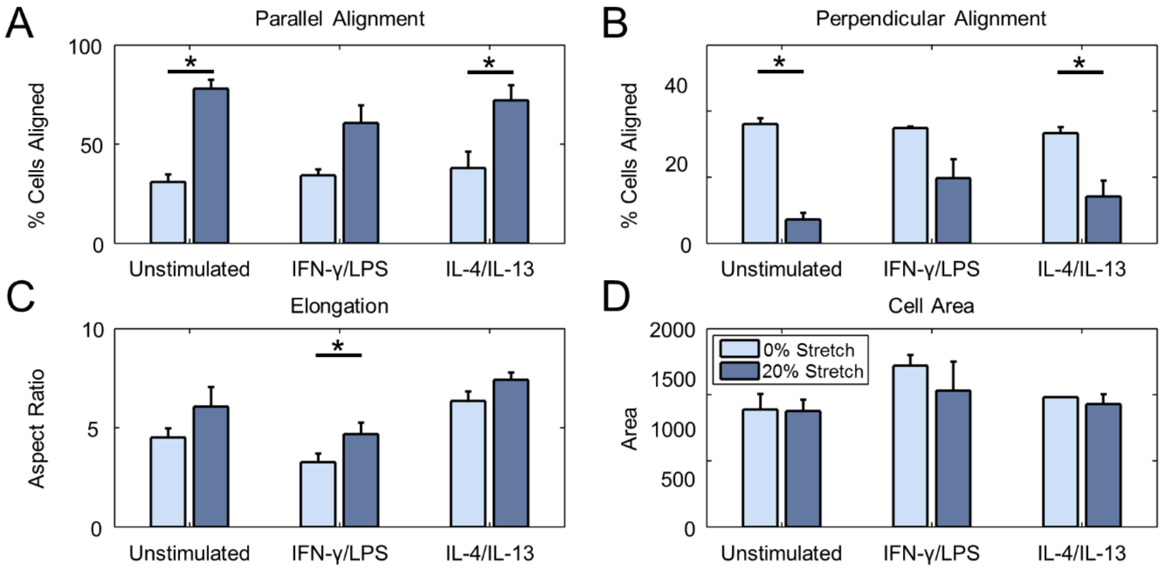

Figure S1: Quantification of cell morphology for macrophages subjected to cyclic uniaxial stretch. Quantification of cell alignment relative to the direction of stretch (A) and perpendicular to the direction of stretch (B), cell aspect ratio or the length of the major axis divided by the length of the minor axis (C), and spread cell area (D) for unstimulated, IFNγ/LPS, and IL4/IL13 stimulated macrophages with no stretch or 20% cyclic uniaxial stretch. Error bars indicate standard deviation for three separate experiments and * $p < 0.05$ when compared to the indicated condition as determined by Student’s t-test.

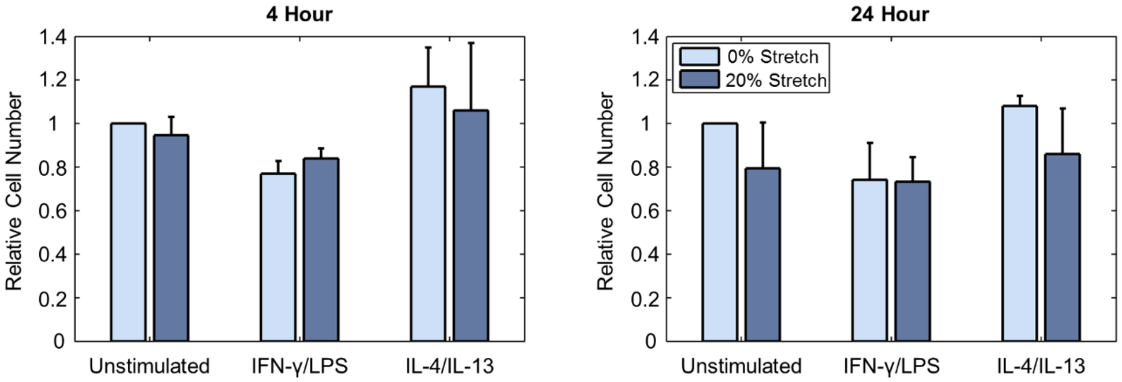

Figure S2: Cyclic mechanical stretch does not affect macrophage cell viability. Quantification of cell viability, as measured by Cyquant assay, following either 4 (left) or 24 (right) h of adhesion, stimulation, and 18 h of stretch. Cell number was normalized to the unstimulated, 0% stretch, condition for each time point. Error bars indicate standard deviation about the mean for three separate experiments.

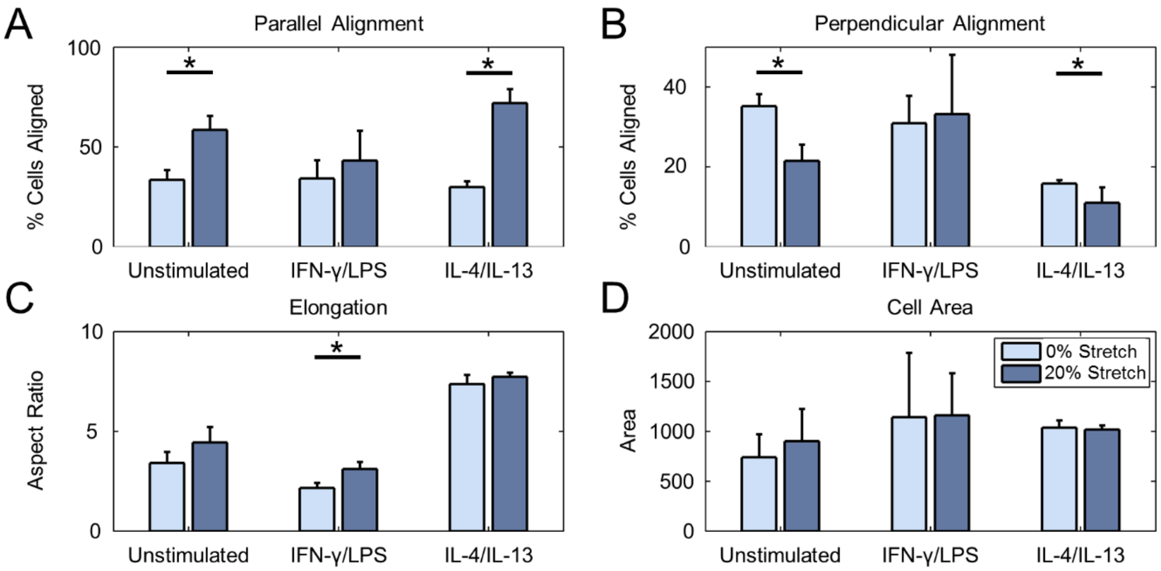

Figure S3: Quantification of morphological parameters for macrophages subjected to cyclic uniaxial stretch after 4 h of adhesion. Quantification of cell alignment relative to the direction of stretch (A) and perpendicular to the direction of stretch (B), as defined by the highlighted regions in Figure 1B, cell aspect ratio or the length of the major axis divided by the length of the minor axis (C), and spread cell area (D) for unstimulated, IFNγ/LPS, and IL4/IL13 stimulated macrophages with no stretch or 20% cyclic uniaxial stretch. Error bars indicate standard deviation for three separate experiments and * $p < 0.05$ when compared to the indicated condition as determined by Student's t-test.
